# Infestation, Community Structure, and Seasonal Dynamics of Chiggers on Small Mammals at a Focus of Scrub Typhus in Northern Yunnan, Southwest China

**DOI:** 10.3390/insects17010031

**Published:** 2025-12-24

**Authors:** Yan Lv, Peng-Wu Yin, Xian-Guo Guo, Rong Fan, Cheng-Fu Zhao, Zhi-Wei Zhang, Ya-Fei Zhao, Lei Zhang

**Affiliations:** Institute of Pathogens and Vectors, Yunnan Provincial Key Laboratory for Zoonosis Control and Prevention, Dali University, Dali 671000, China

**Keywords:** chigger mite, ectoparasite, seasonal fluctuation, rodent, *Leptotrombidium deliense*, *Leptotrombidium scutellare*, *Leptotrombidium imphalum*

## Abstract

Chiggers are common ectoparasites and the exclusive vector of scrub typhus. From November 2020 to October 2021, a 12-month investigation was conducted at Waxi Village of Binchuan County, Yunnan Province of southwest China, a focus of scrub typhus. A total of 217,671 chiggers collected from 1329 small mammal hosts (belonging to 18 species) at the survey site were taxonomically identified as 115 species and 13 genera in the family Trombiculidae with high species diversity. Among the 115 chigger species identified, there were nine vector species. The prevalence (*P_M_* = 69.38%), mean abundance (*MA* = 163.79 mites/per examined host), and mean intensity (*MI* = 236.09 mites/per infested host) were high. Three rodent species, *Rattus andamanensis*, *Apodemus chevrieri*, and *Mus caroli*, were the dominant hosts. Three vector chiggers, *L. deliense*, *L. scutellare,* and *L. imphalum*, were the dominant chiggers, with a constituent ratio (*C_r_*) of 60.72%. The indexes of chigger infestation and community showed varying degrees of monthly and seasonal fluctuations. From August (summer) to September and October (autumn), the Margalef richness index (*R*), Shannon–Wiener diversity index (*H*), and Pielou evenness (*E*) of chiggers were at low levels, but the Simpson dominance index (*D*) was at a high level. The *C_r_* and infestation indexes (*MA*, *MI*) of chiggers were high from August to October in summer and autumn, with abundant vector chiggers (such as *L. deliense* and *L. imphalum*) appearing in these seasons. The species richness of chigger community was highest in winter, but lowest in summer. The similarity of the chigger community was very high between August (summer) and September to October (autumn). The evenness of the chigger community was highest in spring and lowest in summer. In conclusion, small mammals are susceptible to chigger infestation with coexistence of multiple vector chigger species. Most chigger species have a wide range of hosts with low host specificity. The abundant vector chiggers (especially *L. deliense* and *L. imphalum*) in summer and autumn suggests that the surveillance campaign of scrub typhus and vector chiggers should mainly focus on these two seasons at the survey site.

## Simple Summary

Being a common group of ectoparasites of rodents and other small mammals, chiggers (chigger mites) are the exclusive vector of scrub typhus and can also serve as potential vectors of hemorrhagic fever with renal syndrome (HFRS). From November 2020 to October 2021, a 12-month investigation was conducted to illustrate the infestation, community structure and seasonal dynamics of chiggers on small mammals at a focus of scrub typhus in northern Yunnan, southwest China. A total of 217,671 chiggers collected from 1329 small mammal hosts at the survey site (Waxi Village) were taxonomically identified as 115 species and 13 genera in the family Trombiculidae, with high species diversity. Rodents and other sympatric small animals at Waxi Village were susceptible to chigger infestation, with high infestation burdens, and coexistence of multiple vector chiggers. Most chigger species had a wide range of hosts with low host specificity. The chigger community showed a high similarity between August and October in summer and autumn with low species diversity, and a large number of vector chiggers, such as *Leptotrombidium deliense* and *L. imphalum,* appeared in these seasons.

## 1. Introduction

Rodents and other sympatric small mammals are of medical significance, and they can serve as the infectious source and reservoir host of many zoonotic diseases such as plague, murine typhus, scrub typhus, and hemorrhagic fever with renal syndrome (HFRS) [1,2]. Chiggers (chigger mites) usually refer to the larval stage of trombiculid mites (Trombiculidae) with over 3000 species recorded in the world, and they are a common group of ectoparasites on rodents and other small mammals [3,4]. In the life cycle of trombiculid mites, only the larval stage (chigger) is ectoparasitic, while the other stages are all free-living [5,6]. Chiggers are the exclusive vector of scrub typhus, and through their biting activities, *Orientia tsutsugamushi* (Ot) can be transmitted among wild animals (e.g., rodents and other small mammals) and even from wild animals to domestic animals and humans [7,8]. Besides transmitting scrub typhus, some chiggers (e.g., *Leptotrombidium scutellare*) can act as the potential vectors of HFRS [9,10]. Scrub typhus is widespread in China, with the incidence being in continuous rise and the foci gradually expanding in recent years [11,12]. Yunnan Province, located in the southwest of China, is a very important focus of scrub typhus and HFRS. Among the 129 counties in 16 prefectural administrative regions of Yunnan Province, 118 counties in all the 16 prefectural regions have reported cases of scrub typhus [13]. Of the 283,273 cases of scrub typhus reported in mainland China between 2006 and 2023, 84,795 cases from Yunnan Province ranked first, accounting for 29.93% of the total in China [11].

The community study is an important aspect in ecological fields [14,15]. In fact, the seasonal variation patterns of the same group of mites may vary from region to region. To study the seasonal variations in a specific mite group (or a specific species), it is recommended to select a fixed survey site and conduct field investigations once a month for a period of at least one entire year (a 12-month consecutive field investigation). The present study selected Waxi Village of Binchuan County, Dali Prefecture in Yunnan Province of southwest China as the fixed survey site and conducted a consecutive 12-month investigation. The survey site is a focus of scrub typhus and HFRS [16,17]. The study is an attempt to illustrate the infestation, community structure and seasonal dynamics of chiggers in this epidemic focus. The hosts of chiggers involved in this study were mainly rodents (Rodentia) and other sympatric small animals such as shrews (Eulipotyphla) and tree shrews (Scandetia), excluding volant bats (Chiroptera).

## 2. Materials and Methods

### 2.1. Field Investigations

**Figure 1:**
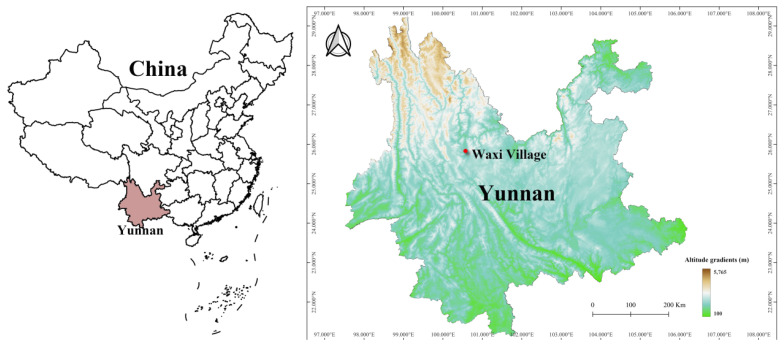
Map of China and Yunnan Province showing the location of the fixed survey site (Waxi Village) in Binchuan County, Yunnan Province of southwest China (2020–2021).

A consecutive 12-month investigation was carried out at Waxi Village of Binchuan County, Dali Prefecture in Yunnan Province of southwest China from November 2020 to October 2021 (Figure 1). The field investigation each month lasted for seven to ten days to ensure a sufficient number of host samples (at least 100 hosts per month). The fixed survey site, Waxi village (25°43′ N, 100°24′ E), belongs to a subtropical area, with a typical subtropical monsoon climate [18].

### 2.2. Collection of Animal Hosts and Chiggers

At the fixed survey site, mouse traps (18 × 12 × 9 cm, Guixi Mousetrap Apparatus Factory, Guixi, Jiangxi, China) were placed in different habitats to capture rodents and other sympatric small mammals in the late afternoon or evening. The microhabitats for the trap placement included residential areas, farmlands, uncultivated lands, shrublands, and riverbanks. Each captured host was separately collected with a white cloth bag in the following morning and then were transported to the laboratory for the collection of chiggers. Each animal host was conventionally anesthetized with ether and placed in a large white square plate to collect chiggers. With the help of magnifier, a lancet or curette was used to scrape chiggers or chigger-like substance from each host’s auricle, external auditory canal opening, groin, perianal area, and perineum, where chiggers often attach. The collected chiggers (including chigger-like substance) from each host were separately placed in a covered centrifuge tube or other covered vial filled with 70% or 75% ethanol for fixation and preservation [19,20]. After the chigger collection, each host was identified to species according to its external morphology (size, shape, and color) and body measurements (body length, body weight, tail length, ear height, hind foot length, etc.) [21,22,23]. The capture and use of animal hosts for the research was officially approved by the Animals’ Ethics Committee of Dali University, approval code: DLDXLL2020-1104, approval date: 4 November 2020.

### 2.3. Specimen-Making and Taxonomic Identification of Chiggers

In the study of chigger community, it is essential to conduct the taxonomic identification of the large number of chiggers collected from the field surveys. As the larval stage of trombiculid mites, chiggers are extremely small and their morphological structure has not yet fully developed. The taxonomic identification of chiggers is technically challenging, which, to some extent, limits the study of chigger community. Before the taxonomic identification, the specimen-making of chiggers must be carried out, and each chigger specimen must be carefully observed and measured under the high power or oil lens of a microscope [5,24]. The chiggers and chigger-like substance collected from the field surveys were rinsed with clean or distilled water two to three times, and then placed under a stereomicroscope to isolate chiggers from other impurities (chigger-like substance). The isolated chiggers were mounted onto glass slides with Hoyer’s fixative medium. After dehydration, drying, and transparent process, each mounted chigger specimen was meticulously observed and measured under the high power or oil lens of a microscope. Under the microscope, each chigger specimen was ultimately identified to species according to relevant taxonomic books, the literature, and taxonomic keys [6,25,26,27].

### 2.4. Basic Statistics of Chigger Infestation and Community

The conventional statistical analysis was conducted on the species composition and constituent ratio (*C_r_*) of hosts and chiggers. Based on the *C_r_* of different species within the community, the dominant species of hosts and chiggers at the survey sites were determined. The infestation status of chiggers on hosts was reflected by the prevalence (*P_M_*), mean abundance (*MA*), and mean intensity (*MI*). The *P_M_* reflects the frequency of chigger infestation on the hosts. The *MA* and *MI* indicate the intensity of chigger infestation on the hosts, with *MA* standing for the average chiggers per examined host and *MI* for the average chiggers per infested host [28,29,30]. The significance test for *C_r_* and *P_M_* was conducted using the chi-square test, while the non-parametric rank-sum test was used for *MA* and *MI*.

The following formulas were used to calculate the species richness (*S*), Margalef richness index (*R*), Shannon–Wiener diversity index (*H*), Pielou evenness (*E*), and Simpson dominance index (*D*) [31]. Of these community indexes, *S* is actually the total number of all species within the community.

(1) $$S=\sum S_{i}$$

(2) $$R=\frac{S-1}{\ln N}$$

(3) $$H=-\sum_{i=1}^{S}\left(\frac{N_{i}}{N}\right)\ln\left(\frac{N_{i}}{N}\right)$$

(4) $$E=\frac{H}{\ln S}$$

(5) $$D={\sum_{i=1}^{S}\left(\frac{N_{i}}{N}\right)}^{2}$$

In the above formulae, *S_i_* = species *i* in the community; *N_i_* = the number of a certain species in the community; *N* = the total number of all the species; and ln = natural logarithm.

### 2.5. Analysis of Species Abundance and Species-Sample Relationship

In the analysis of species abundance distribution and species-sample relation of chigger community, all the identified chigger species from small mammal hosts at the survey site were regarded as a chigger community unit. To illustrate the species abundance distribution of chigger community on small mammals, a semi-logarithmic rectangular system was established. The *X*-axis (indicating individuals of chiggers) was labeled with log intervals based on log_3_M, and the *Y*-axis (representing the number of chigger species) was marked with arithmetic scales. Based on the calculation of fitting goodness (*R*^2^), Preston’s lognormal model was used to fit the theoretical curve of species abundance distribution [3,20]. The model calculation was accomplished using Excel Office 2019 and the “sads package” of R version 4.4.3. The parameter optimization method employed was the maximum likelihood estimation method. The expected total number of chigger species (*S_T_*) and the likely missed species (*S_M_*) in the field survey were approximately estimated according to the formulae:

(6) $$S(R)=S_{0}e^{-[a(R-R_{0}){]}^{2}}(\mathrm{Preston’s lognormal model})$$

(7) $$S_{T}=\left(S_{0}\sqrt{\pi }\right)/\alpha$$

(8) $$S_{M}=S_{T}-S_{A}$$

In the above formulae, *S*(*R*) = the expected theoretical number of chigger species at *R*-th log interval, *R*_0_ = the mode log interval with the highest number of species, *S*_0_ = the number of species at the log interval *R*_0_, α = the spread constant of distribution, and *S_A_* = the number of chigger species actually collected in the field survey. The value of α was determined based on the best fitting goodness, *R*^2^ [3,20].

In the analysis of species-sample relation, all the 1329 small mammal hosts captured in the field survey were randomly numbered and grouped, with each group consisting of 100 individual hosts. The number of chigger species collected from each group of small mammal hosts was counted. The number of host samples (host individuals) was labeled at the *X*-axis and the number of chigger species was marked at the *Y*-axis, and then the species-sample curve was created in a rectangular coordinate system [32].

### 2.6. Analysis of Chigger-Host and Chigger-Chigger Relationship

In the analysis of chigger-host and chigger-chigger relationship, the main chigger species with large individuals within the chigger community were selected as the objects. The bipartite network analysis was used to analyze the chigger-host relationship, the bilateral relationship between the main chigger species and their hosts [3]. In the visualized diagram of the bipartite network analysis, the widths of color bands (or patches) represent the constituent ratios of the main chigger species and their corresponding small mammal hosts. Pearson correlation coefficient was used to calculate the interspecific correlation between any two of the main chigger species (the chigger-chigger relationship), and the results were visualized using a correlation heatmap [3].

### 2.7. Analysis on Seasonal Dynamics of Chigger Community

Based on the calculation of the species composition, *C_r_*, infestation indexes (*P_M_*, *MA*, *MI*) and community indexes (*R*, *H*, *E*, *D*) of chiggers in each month, the monthly variation curves for these indexes were separately created in a rectangular coordinate system to reflect their seasonal fluctuations. The seasonal dynamics of chigger community was visualized using the abundance rank curve, non-metric multidimensional scaling ordination (NMDS) combined with PERMANOVA (Adonis analysis) (NMDS + PERMANOVA), Venn diagram, and Pareto chart [33,34,35].

## 3. Results

### 3.1. Species Composition of Small Mammal Hosts and Chiggers

**Figure 2:**
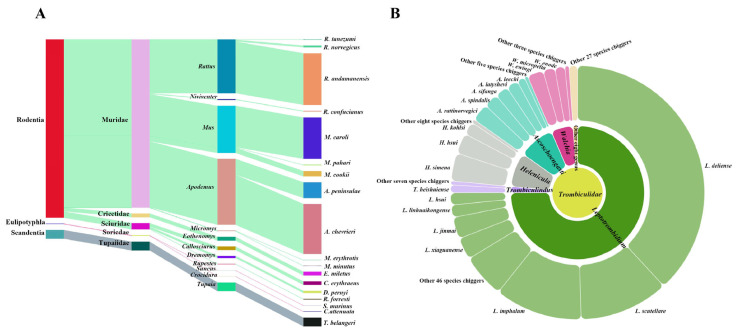
Visualization of host and chigger mite distribution. Annotation: (**A**) The constituent ratios (*C_r_*) of small mammal hosts (1329 individuals) at different orders, families, genera, and species. The shade width indicates the constituent ratio of hosts. (**B**) The constituent ratios (*C_r_*) of chiggers (217,671 individuals) at different taxonomic levels (family, genera, and species).

A total of 1329 rodents and other sympatric small mammals captured were identified as 18 species, 12 genera, and 5 families in three orders (Rodentia, Eulipotyphla, Scandetia). Of the three orders, the number of Rodentia (rodents) was the most abundant, with a total *C_r_* of 95.03% (Figure 2A). Of the 18 host species, 3 rodent species were the most numerous with total *C_r_* = 76.30%, and they were *Rattus andamanensis*, *Apodemus chevrieri* and *Mus caroli* (Table 1, Figure 2A).

A total of 217,671 chiggers collected from 1329 hosts were taxonomically identified as 115 species, 13 genera and 2 subfamilies in the family Trombiculidae (Table 2). A total of 5454 chiggers could not be accurately identified to species, and they were regarded as “unidentified specimens” because of the structural damage, dirt covering, unclear structure, or suspected new species; these unidentified specimens were not included in the data statistics of the study. Of 18 small mammal host species, 2 rat species (*R. andamanensis* and *A. chevrieri*) harbored the largest numbers of chigger species and individuals (Table 1). At the subfamily level, 94.11% of chigger individuals came from the subfamily Trombiculinae. At the genus level, 74.99% of chiggers belonged to the genus *Leptotrombidium*. At the species level, *L. deliense*, *L. scutellare,* and *L. imphalum* accounted for 60.72% of the total 115 chigger species (Table 2, Figure 2B).

### 3.2. Infestation of Vector Chiggers

The overall infestation indexes of chiggers on all hosts throughout the 12 months at the survey site were as follows: *P_M_* = 69.38%, *MA* = 163.79 mites/per examined host, *MI* = 236.09 mites/per infested host (Table 3). Of the 115 chigger species identified, 9 species can serve as vectors or potential vectors of scrub typhus and HFRS. These nine vector chiggers were: *L*. *deliense*, *L. scutellare*, *L. imphalum*, *L. linhuaikongense*, *L. rubellum*, *L. apodemi*, *L. intermedium*, *L. kaohuense,* and *Walchia pacifica* (Table 2). Of the nine vector species, *L. deliense*, *L. scutellare* and *L. imphalum* were also three dominant chigger species, accounting for 60.72% of the total 115 chigger species (Table 2). The infestation indexes of *L. deliense* were the highest (Table 3).

### 3.3. Infestation and Community Indexes of Chiggers on Main Hosts

Of the 18 host species, *R. andamanensis*, *A. chevrieri,* and *M. caroli* were 3 dominant host species (Table 1). Among these three dominant host species, the infestation indexes of chiggers on *R. andamanensis* were the highest. The species richness of chigger community on *R. andamanensis* was higher than that of other two dominant host species. The *R* and *D* of chigger community on *A. chevrieri* were higher than those on *R. andamanensis* and *M. caroli*. The *H* and *E* of chigger community on *M. caroli* were higher than those on *R. andamanensis* and *A. chevrieri* (Table 4).

### 3.4. Species Abundance Distribution and Species-Sample Relationship

**Figure 3:**
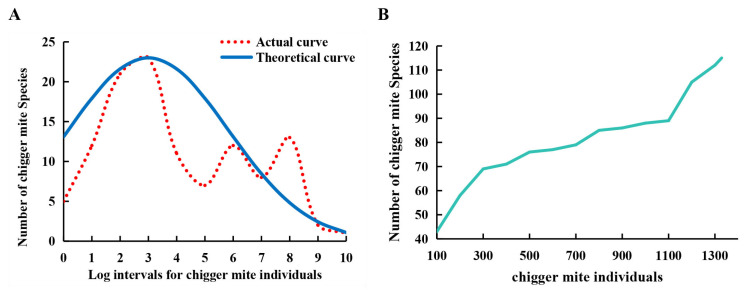
The species-abundance and species-sample curves of chigger community. Annotation: (**A**) The species-abundance curve. (**B**) The species-sample curve.

Based on Preston’s lognormal model, the species abundance distribution of chigger community in the study area was successfully fitted by the lognormal distribution with α = 0.25 and *R*^2^ = 0.99 (Figure 3A). From the theoretical curve tendency of species abundance distribution, it could be found that a very small number of species were the dominant species with a large number of individuals (>9842) in the chigger community. The vast majority of chigger species were those with a relatively small number of individuals, followed by the rare species with few individuals (Table 5, Figure 3A). On the basis of the curve fitting of species abundance distribution, the expected total number (*S_T_*) of chigger species in the chigger community was estimated to be 163, 48 more than the number of species actually collected. The curve of species-sample relationship showed that the number of chigger species gradually increased with the increase in host individuals (Figure 3B).

### 3.5. Chigger-Host and Chigger-Chigger Relationship

**Figure 4:**
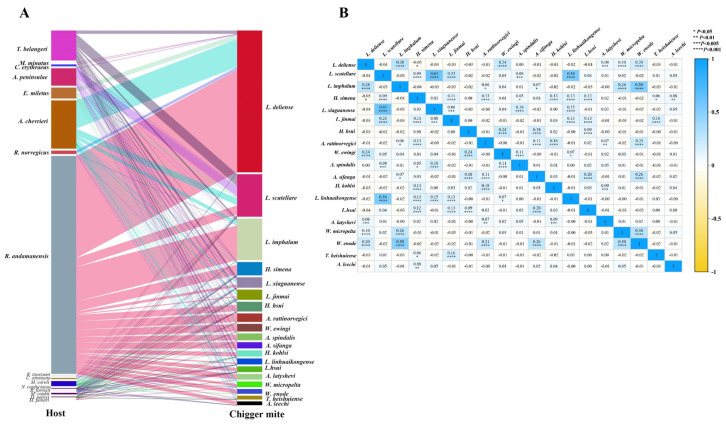
Bilateral relationship between chiggers and their hosts and interspecific relationship of main chigger species. Annotation: (**A**) The bipartite diagram for visualizing the relationships between 19 main chigger species and their corresponding small mammal hosts. (**B**) The correlation heatmap for visualizing the interspecific relationships of 19 main chigger species.

The 19 main chigger species with more than 2000 individuals per species were selected to analyze the bilateral relationship between chiggers and their hosts (chigger-host relationship). These 19 main chigger species had 199,061 individuals, accounting for 91.45% of the total chiggers, which included the 3 dominant chigger species, *L. deliense*, *L. scutellare* and *L. imphalum*. The 19 main chigger species corresponded to 16 different host species, excluding 2 host species (one *Suncus murinus* and one *Micromys erythrotis*) without harboring any of the 19 main chigger species. The bipartite diagram for visualizing the chigger-host relationship showed that one host species could harbor several different chigger species, and one chigger species could parasitize multiple host species with low host specificity. Of the 19 main chigger species, *L. scutellare* and *Helenicula simena* were, respectively, found on 14 and 13 species of hosts with the widest host rage and the lowest host specificity. The other 4 chigger species also had very low host specificity, being found on 12 host species, and they are *L. jinmai*, *L. xiaguanense*, *L. hsui*, and *Helenicula hsui*. *Ascoschoengastia latyshevi* had the narrowest host range, but it still parasitized 4 host species, *R. andamanensis*, *A. chevrieri*, *Dremomys pernyi*, and *Tupaia belangeri* (Figure 4A).

Pearson correlation coefficient (*r*) was used to analyze the interspecific relationships between any 2 of the 19 main chigger species. The results showed that a relatively high positive correlation (*r* ≥ 0.5) existed between *L. scutellare* and *L. xiaguanense* (*r* = 0.61, *p* < 0.001), between *L. scutellare* and *L. linhuaikongense* (*r* = 0.54, *p* < 0.001), and between *L. imphalum* and *W. enode* (*r* = 0.50, *p* < 0.001). For most main chigger species, however, only a slight positive or negative correlation existed between any two of them (*r* < 0.5, *p* < 0.05) (Figure 4B).

### 3.6. Seasonal Dynamics of Chigger Infestation and Community

**Figure 5:**
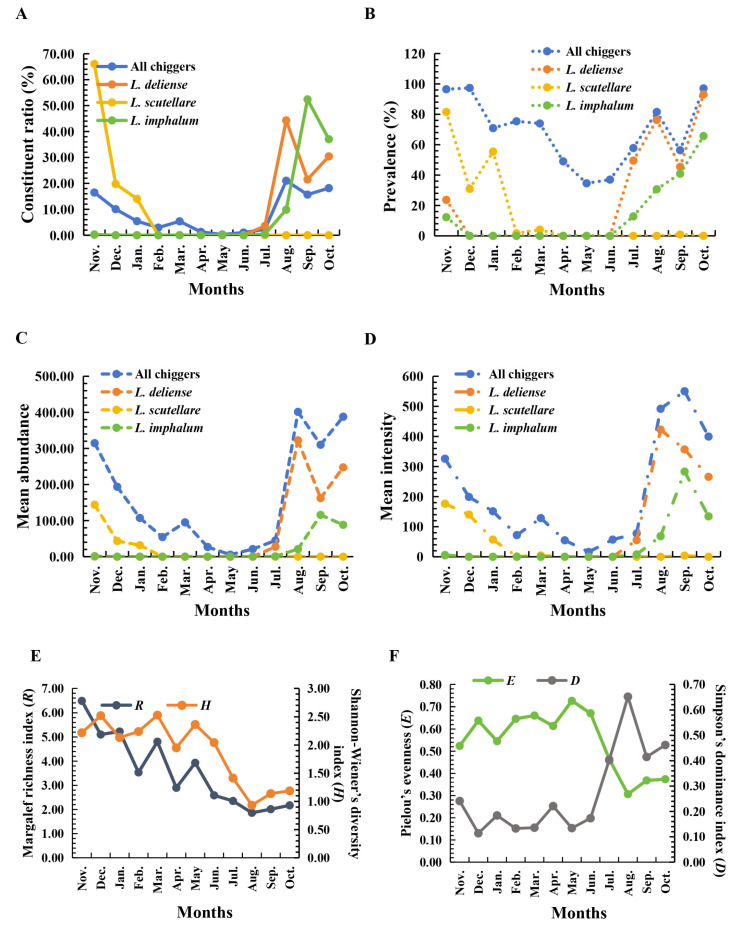
Seasonal fluctuations of infestation indexes of all chiggers and three dominant chigger species (*L. deliense*, *L. scutellare,* and *L. imphalum*) and community indexes of chiggers. Annotation: (**A**) monthly fluctuation of constituent ratios (*C_r_*) of chiggers; (**B**) monthly fluctuation of prevalence (*P_M_*) of chiggers; (**C**) monthly fluctuation of mean abundance (*MA*) of chiggers; (**D**) monthly fluctuation of mean intensity (*MI*) of chiggers; (**E**) monthly fluctuation of Margalef richness index (*R*) and Shannon–Wiener diversity index (*H*) of chigger community; (**F**) and monthly fluctuation of Pielou evenness (*E*) and Simpson dominance index (*D*) of chigger community.

In August (summer) and September to October (autumn), the *C_r_* and overall infestation indexes (*P_M_*, *MA*, *MI*) of chiggers were high, with *C_r_*, *MA*, and *MI* reaching their peaks between August and October. From August to October in summer and autumn, the *R*, *H,* and *E* of chigger community were at low levels, while the *D* was at a high level (Table 6; Figure 5). Of the three dominant chigger species, *L. deliense* and *L. imphalum* were very abundant from August to October in summer and autumn, with their *C_r_* and infestation indexes (*P_M_*, *MA*, *MI*) being relatively high or even reaching their peaks. *Leptotrombidium scutellare*, however, mainly appeared in November at the end of autumn and December at the beginning of winter, with its *C_r_* and infestation indexes (*P_M_*, *MA*, *MI*) reaching their peaks in November at the end of autumn (Figure 5A–D).

**Figure 6:**
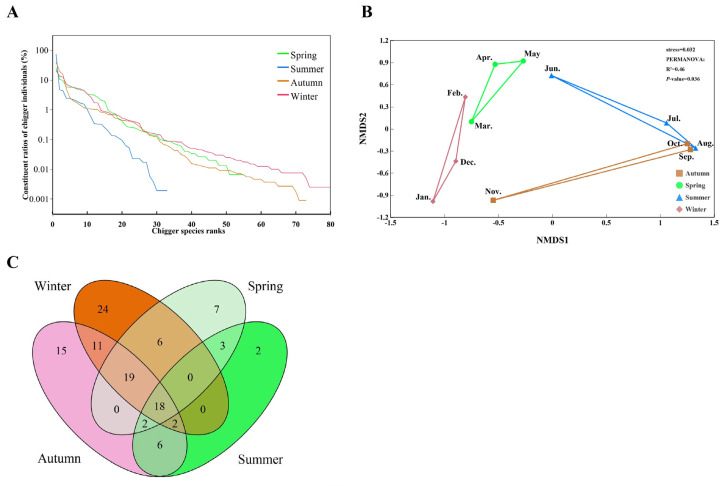
The visualization of seasonal dynamics of chigger community on small mammals in different seasons. Annotation: (**A**) Species rank abundance curves of chigger community in different seasons. The chigger species ranks from 1 to 80 on the horizontal axis (*X*-axis) represent the most dominant chigger species to the rarest chigger species. (**B**) The dimension-reduced ordination of chigger community in different seasons by non-metric multidimensional scaling ordination (NMDS) and PERMANOVA (Adonis). (**C**) The Venn diagram for visualizing the species distribution of chigger community in different seasons.

**Figure 7:**
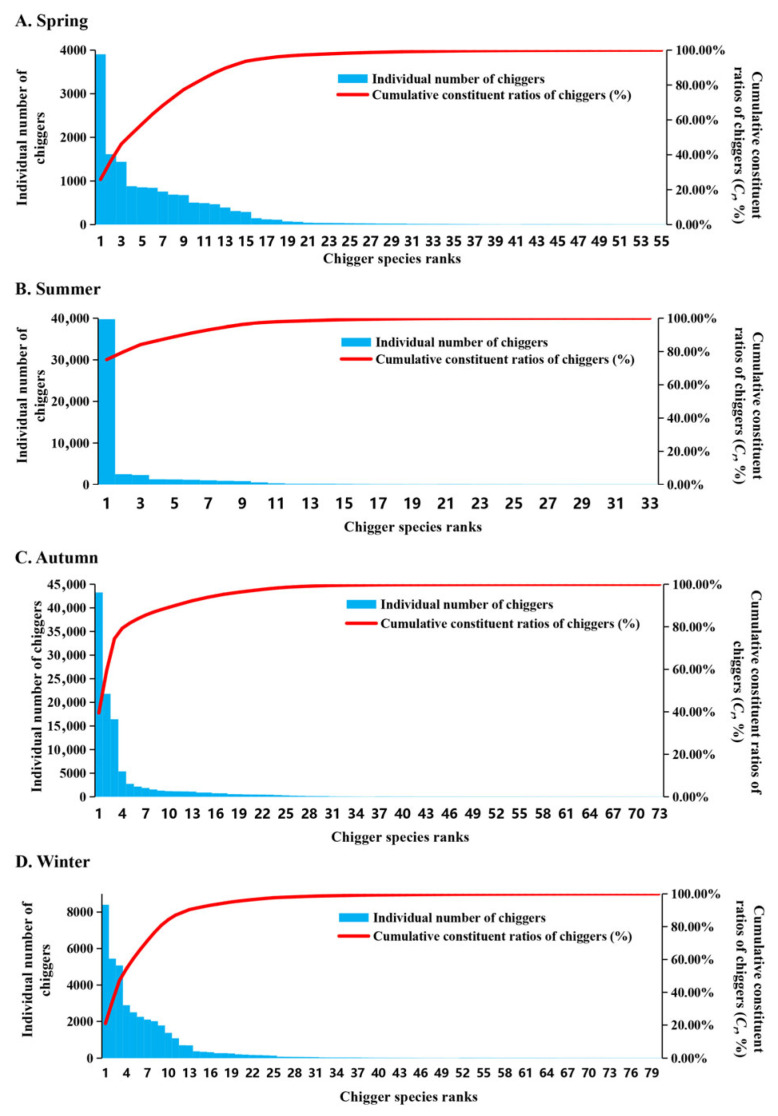
The Pareto chart for visualizing the contribution of main chigger species in different seasons to the cumulative constituent ratio (*C_r_*) of chigger community on small mammals. Annotation: The chigger species ranks from 1 to 80 on the horizontal axis (*X*-axis) represent the most dominant chigger species to the rarest chigger species.

The results of the species rank abundance curve showed that the extension length of the curve in the horizontal direction was the longest in winter and the shortest in summer, indicating that the species richness of chigger community was the highest in winter and the lowest in summer. In the vertical direction, the slope of the species rank abundance curve was the flattest in spring and the steepest in summer, indicating that the evenness of chigger community was the highest in spring and the lowest in summer (Figure 6A). In a two-dimensional coordinate system, the results showed that the similarity of chigger community was very high in August (summer) and September to October (autumn), with the positions of these three months being very close (Figure 6B). The Venn diagram visually reflected the overall distribution of chigger species in the four seasons of spring, summer, autumn, and winter, as well as the distribution of endemic species and overlapping species in different seasons (Figure 6C). For instance, the number of chigger species in spring, summer, autumn, and winter were 55, 33, 73, and 80, respectively. The endemic species in the four seasons were 7, 2, 15, and 24, respectively, with the most endemic species in winter (24 species) and the least in summer (only 2 species). There were 18 chigger species that were distributed in all four seasons, and 19 chigger species that were overlapped in spring, autumn and winter (Figure 6C). The Pareto chart was a visualization based on the Pareto analysis model, reflecting the contribution of the main chigger species in different seasons to the cumulative *C_r_* of chigger community, all chiggers (Figure 7). The results showed that 80.67% of the cumulative *C_r_* of chigger community was mainly contributed by ten chigger species in spring, 79.84% of *C_r_* contributed by two chigger species in summer, 79.28% of *C_r_* contributed by four chigger species in autumn, and 80.91% of *C_r_* contributed by nine chigger species in winter (Figure 7).

## 4. Discussion

### 4.1. Medical Significance of the Chigger Study

The survey site of the present study, Waxi Village of Bichuan County, Dali Prefecture, Yunnan Province of southwest China, is not only an important focus of scrub typhus and HFRS, but also a hot tourist place in China [16,38,39]. Therefore, it is of great medical significance to study chiggers on rodents and other sympatric small animals in this epidemic area. Globally, at least 12 million people were infected with scrub typhus each year [40,41]. In recent years, the incidence of scrub typhus has been increasing in some endemic areas or countries, including China [10,42,43,44]. A total of 69,246 cases of scrub typhus were reported in Yunnan Province from 2013 to 2022, with 14.58/100,000 of the average annual incidence [45]. Yunnan Province (especially Dali Prefecture) is one of main tourist regions in China, and scrub typhus has become one of the most important infectious diseases threatening tourists’ health [45]. In addition to the widespread prevalence of scrub typhus, HFRS is also widely distributed in Yunnan Province, and the epidemic situation is especially severe in the central and northwestern regions of Yunnan (including Dali Prefecture). The incidence of HFRS in Dali Prefecture has been high in recent years, with HFRS cases accounting for 54.47% (999/1834) of the total in Yunnan Province from 2012 to 2020 [16,46].

The results of the present study indicate that rodents and other sympatric small animals were highly susceptible to chigger infestation at the survey site, with abundant species and heavy infestation burden found on their body surface. Previous studies have shown that many chigger species prefer to breed in environments with high temperature and humidity. A warm and humid climate is conducive to the growth and reproduction of many chigger species [47,48]. Waxi Village is located in a low-latitude subtropical area with warm and humid climate. This might be an important reason for rodents and other sympatric small mammals to be infested with abundant chiggers with high species diversity and heavy infestation burdens in the survey site. The heavy infestation burdens and low host specificity would be conducive to the transmission of pathogens of zoonotic diseases such as scrub typhus and HFRS among different rodents and other wild animal hosts at this site and nearby areas, and even from wild animals to domestic animals and humans.

### 4.2. Infestation and Coexistence of Multiple Vector Chiggers

Although there have been over 3000 chigger species recorded globally, not all of them can effectively transmit scrub typhus. It has been confirmed that the majority of effective vector species belong to the genus *Leptotrombidium* [4,10]. In China, it has been proved that there are six main vectors and more than ten secondary or potential vectors of scrub typhus, and most of them are *Leptotrombidium* species [36,49]. In the present study, *L. deliense*, *L. scutellare* and *L. imphalum* were not only the dominants at the survey site, but also the important vector species. *Leptotrombidium deliense* and *L. scutellare* are the most important two of the six main vectors of scrub typhus in China, and *L. imphalum* is an important potential vector of the disease [9,37,50]. In addition, *L. scutellare* can serve as the potential vector of HFRS as well [9,37,50]. In China, many previous studies have shown that *L. deliense* is the dominant chigger species and the most important vector of scrub typhus in the vast territory south of the Yangtze River, including Yunnan and some other regions of southwest China [51,52], and the results of the present study are consistent with previous studies. There were nine vector species coexisting at the survey site, and most chigger species had a wide range of hosts with low host specificity. The occurrence of a large number of *L. deliense*, *L. scutellare,* and *L. imphalum* at Waxi, together with the coexistence of other vector species and low host specificity, would increase the potential risk of the transmission of zoonotic diseases such as scrub typhus and HFRS, and the persistence of the epidemic foci.

### 4.3. Species Abundance and Species-Sample Relationship

The species abundance distribution of a community is used to illustrate the relationship between the number of species and individuals in a certain community, which can reflect the proportion structure of dominant, common, and rare species in the community [32,53]. In the present study, the species abundance distribution was successfully fitted by Preston’s lognormal distribution model (Figure 3A). The result indicates that the species abundance of chigger community conforms to lognormal distribution, with very few dominant species having a large number of individuals, and the majority of common species having a relatively small number of individuals [3,32]. In ecological research, it is an important issue to estimate the expected total number of species in a certain community [54,55]. There are a few methods to roughly estimate the expected total species, and the method used in the present study is one of them [3,20]. Based on the fitting of the theoretical curve of species abundance distribution, the expected total species (*S_T_*) of chigger community at the survey site was roughly estimated to be 163 species, 48 more than the 115 species actually identified (*S_A_*), implying that approximately 48 rare chigger species might have been missed in the actual field investigation. In a community with a lognormal distribution of species abundance, there are some rare species with very few individuals (even one or two individuals), and these rare species are often missed in a certain field investigation [3,20].

The species-sample relationship is used to illustrate the relationship between the sample size (or plot) and the number of species within a certain community [32]. The result showed that the number of chigger species gradually increased with the increase in host samples (Figure 3B), suggesting that it is impossible to find all species in a single field investigation and that more species would be found if host samples continued to increase. The result of the species-sample relationship is consistent with the estimation result of the expected total species, with approximately 48 rare chigger species not being found in the present field investigation.

### 4.4. Host-Chigger and Chigger-Chigger Relationships

The results of the host-chigger relationship showed that one host species could harbor several different chigger species, and one chigger species could parasitize multiple host species with low host specificity. As one of the dominant and vector chigger species, *L. scutellare* had the widest host selection with lowest host specificity (Figure 2A). The low host specificity of chiggers would facilitate the circulatory transmission of some zoonotic pathogens such as *O. tsutsugamushi* and Hantavirus among different host animals, thereby increasing the potential risk of transmission of the corresponding zoonotic diseases (scrub typhus and HFRS) among different hosts and the persistence of their foci [5,10].

The results of the chigger-chigger relationship showed that a relatively high positive correlation (*r* ≥ 0.5) existed between some pairs of main chigger species with statistical significance (*p* < 0.001) (Figure 4B). The results were consistent with some previous reports [56,57], suggesting that some chigger species have a tendency to parasitize the same host species without much interspecific competition or mutual repulsion.

### 4.5. Seasonal Fluctuation of Chigger Infestation and Community

Since chiggers are the exclusive vector of scrub typhus and potential vector of HFRS, the seasonal fluctuations of chiggers would undoubtedly affect the seasonal distribution of these mite-borne diseases. Therefore, the investigation on the seasonal fluctuation of vector chiggers would be conductive to the surveillance and control of scrub typhus and HFRS [24,58]. The results of the present study showed that both the infestation indexes and community indexes of chiggers exhibited varying degrees of monthly and seasonal fluctuations (Table 6; Figure 5). The seasonal fluctuation tendency of overall chigger infestations of all chigger species throughout the 12 months were basically consistent with that of the three dominant vector chigger species (Figure 5A–D), suggesting that the seasonal variation in these dominant chiggers (especially *L. deliense* and *L. imphalum*) determine that of the whole chigger community. The large numbers of the three vector chiggers (*L. deliense*, *L. imphalum,* and *L. scutellare*) occurring in summer and autumn seasons would probably increase the potential risk of scrub typhus outbreaks in these two seasons. Therefore, the surveillance campaign of scrub typhus and vector chiggers should mainly focus on these two seasons at the survey site.

High species diversity and evenness with relatively few dominant species in a certain community usually reflect the community’s complexity. A complex community with many species may inhibit the outbreak of vectors, and a simple community without abundant species may be conductive to the outbreak of vectors [5,59,60]. The results of the present study indicate that species diversity and evenness of the chigger community are much lower in summer and autumn seasons than in winter and spring seasons. In summer and autumn seasons, only three vector chigger species (*L. deliense*, *L. imphalum,* and *L. scutellare*) were the dominant species. Due to the simple community structure without many chigger species in summer and autumn seasons, the outbreak probability of vector chiggers would be much higher in summer and autumn than in winter and spring at the survey site.

## 5. Conclusions

At the survey site, Waxi village of Binchuan County, Dali Prefecture, Yunnan Province of southwest China, where scrub typhus is prevalent, rodents and other sympatric small animals are susceptible to chigger infestation, with high infestation burdens, high species diversity, and the coexistence of multiple vector chiggers. The main hosts of chiggers are three rodent species, *R. andamanensis*, *A. chevrieri,* and *M. caroli*. Three vector chigger species, *L. deliense*, *L. scutellare,* and *L. imphalum*, are the dominant species of the chigger community. Most chigger species have a wide range of hosts with low host specificity. One host species can harbor multiple chigger species, and one chigger species can parasitize multiple host species. The infestation and community indexes of chiggers have seasonal fluctuations. In seasonal fluctuation patterns, *L. deliense* and *L. imphalum* (summer-autumn type) are very different from *L. scutellare* (autumn-winter type). The chigger community shows a high similarity between August and October in summer and autumn with low species diversity, and a large number of vector chiggers such as *L. deliense* and *L. imphalum* appear in these seasons. The occurrence of abundant vector chiggers (especially *L. deliense* and *L. imphalum*) in summer and autumn suggests that the surveillance campaign of scrub typhus and vector chiggers should mainly focus on these two seasons at the survey site.

## Figures and Tables

**Table 1:** Collection and identification of small mammal hosts and their ectoparasitic chiggers at Waxi Village of Binchuan County, Yunnan Province of southwest China (2020–2021).

Host Species	Individuals of Hosts		Species and Individuals of Chiggers		
	Individuals	*C_r_* (%)	Species	Individuals	*C_r_* (%)
*Rattus andamanensis*	366	27.54	83	135,662	62.32
*Apodemus chevrieri*	355	26.71	73	31,274	14.37
*Mus caroli*	293	22.05	38	3332	1.53
*Apodemus peninsulae*	112	8.43	47	11,720	5.38
*Tupaia belangeri*	62	4.67	50	20,745	9.53
*Mus cookii*	38	2.86	16	1043	0.48
*Eothenomys miletus*	27	2.03	37	7951	3.65
*Callosciurus erythraeus*	25	1.88	18	1167	0.54
*Dremomys pernyi*	16	1.20	17	766	0.35
*Rattus norvegicus*	13	0.98	13	2070	0.95
*Niviventer confucianus*	6	0.45	33	849	0.39
*Rupestes forresti*	4	0.30	10	98	0.05
*Rattus tanezumi*	3	0.23	7	108	0.05
*Mus pahari*	3	0.23	7	29	0.01
*Crocidura attenuata*	3	0.23	10	826	0.38
*Micromys erythrotis*	1	0.08	0	0	0.00
*Micromys minutus*	1	0.08	1	31	0.01
*Suncus murinus*	1	0.08	0	0	0.00
Tatal	1329	100.00	115	217,671	100.00

**Table 2:** Taxonomic identification of chiggers on small mammal hosts at Waxi Village of Binchuan County, Yunnan Province of southwest China (2020–2021).

Taxonomic Taxa of Chigger Mites	Collected Species and Individuals of Chigger Mites (The Figures Enclosed in Parentheses Are the Collected Individuals for Each Mite Species)
Trombiculidae	217,671 individuals, 115 species, 13 genera, 2 subfamilies
Trombiculinae	204,852 individuals, 92 species, 9 genera
*Leptotrombidium*	Leptotrombidium deliense * (83008), L. scutellare * (24839), L. imphalum * (24313), *L. xiaguanense* (6098), *L. jinmai* (5972), L. linhuaikongense * (3474), *L. hsui* (3339), *L. suense* (1838), *L. eothenomydis* (1356), *L. densipunctatum* (1347), *L. shuqui* (1109), *L. fujianense* (1096), *L. ejingshanense* (880), *L. yongshengense* (796), *L. apodevrieri* (635), *L. muntiaci* (562), L. rubellum * (541), *L. baoshui* (482), *L. gemiticulum* (275), *L. kunmingense* (195), *L. chuanxi* (174), L. apodemi * (121), *L. sinotupaium* (114), *L. qiui* (102), *L. rusticum* (92), *L. gongshanense* (82), *L. zhongdianense* (54), *L. qujingense* (49), *L. yunlingense* (33), *L. bishanense* (28), *L. bambicola* (24), *L. pavlovskyi* (22), *L. biluoxueshanense* (19), *L. laxoscutum* (16), *L. hiemalis* (15), *L. dianchi* (14), *L. rupestre* (14), *L. deplanoscutum* (13), *L. linji* (13), *L. shuyui* (10), *L. yulini* (9), *L. dichotogalium* (8), *L. longchuanense* (6), *L. myotis* (6), *L. caudatum* (6), *L. lianghense* (6), L. intermedium * (5), *L. wangi* (5), L. kaohuense * (4), *L. sinicum* (3), *L. alpinum* (1), *L. taiyuanense* (1), *L. xishani* (1)
*Trombiculindus*	*Trombiculindus heishuiense* (2226), *T. bambusoides* (812), *T. hunanye* (126), *T. sanxiaensis* (33), *T. yunnanus* (26), *T. nujiange* (25), *T. hylomydis* (5), *T. spinifoliatus* (1)
*Muritrombicula*	*Muritrombicula dali* (3)
*Microtrombicula*	*Microtrombicula nadchatrami* (71)
*Helenicula*	*Helenicula simena* (7782), *H. hsui* (5700), *H. kohlsi* (3639), *H. yunnanensis* (864), *H. litchia* (60), *H. lanius* (35), *H. abaensis* (22), *H. globularis* (14), *H. rectangia* (3), *H. miyagawai* (3), *H. olsufjevi* (2)
*Doloisia*	*Doloisia taishanensis* (6)
*Cheladonta*	*Cheladonta micheneri* (812), *C. ikaoensis* (3), *C. deqinensis* (2)
Ascoschoengastia	*Ascoschoengastia rattinorvegici* (4961), *A. spindalis* (3993), *A. sifanga* (3650), *A. latyshevi* (3287), *A. leechi* (2218), *A. crassiclava* (689), *A. yunnanensis* (205), *A. petauristae* (16), *A. aliena* (5), *A. yunwui* (5)
*Herpetacarus*	*Herpetacarus callosciuri* (195), *H. hastoclavus* (64), *H. aristoclavus* (38), *H. fukienensis* (26)
Gahrliepiinae	12,819 individuals, 23 species, 4 genera
*Walchia*	*Walchia ewingi* (4487), *W. micropelta* (3281), *W. enode* (2794), *W. kor* (940), *W. sheensis* (175), W. pacifica * (1)
*Schoengastiella*	*Schoengastiella ligula* (44)
*Gahrliepia*	*Gahrliepia miyi* (909), *G. madun* (37), *G. myriosetosa* (31), *G. agrariusia* (25), *G. chekiangensis* (18), *G. tenuiclava* (15), *G. octosetosa* (12), *G. megascuta* (10), *G. pintanensis* (8), *G. linguipelta* (8), *G. yunnanensis* (7), *G. longipedalis* (5), *G. latiscutata* (3)
*Chatia*	*Chatia maoyi* (4), *C. hertigi* (3), *C. alpine* (2)

Annotation: The species marked with “*” are vectors or potential vectors of scrub typhus and HFRS [36,37].

**Table 3:** Infestation indexes of dominant chigger species.

Dominant Chigger Species	No. of Hosts		No. and Constituent Ratios (*C_r_*) of Chiggers		Infestation Indexes of Chiggers		
	Hosts Examined	Hosts Infested	No.	*C_r_*, %	*P_M_*	*MA*	*MI*
*L. deliense*	1329	313	83,008	38.13	23.55	62.46	265.20
*L. scutellare*	1329	197	24,839	11.41	14.82	18.69	126.09
*L. imphalum*	1329	175	24,313	11.17	13.17	18.29	138.93
Total of three dominant mite species	1329	499	132,160	60.72	37.55	99.44	264.85
115 chigger species	1329	922	217,671	100.00	69.38	163.79	236.09

Annotation: *P_M_* = prevalence, *MA* = mean abundance, and *MI* = mean intensity.

**Table 4:** Infestation and community indexes of chiggers on three dominant host species.

Dominant Host Species	Infestation Indexes of Chiggers			Indexes of Chigger Communities				
	*P_M_* (%)	*MA*	*MI*	*S*	*R*	*H*	*E*	*D*
*R. andamanensis*	95.63	370.66	387.61	83	6.94	2.29	0.52	0.23
*A. chevrieri*	65.92	88.10	133.65	73	6.96	1.94	0.45	0.30
*M. caroli*	38.57	11.37	29.49	38	4.56	2.35	0.65	0.14

Annotation: *S* = species richness, *R* = Margalef richness index, *H* = Shannon–Wiener diversity index, *E* = Pielou evenness, and *D* = Simpson dominance index.

**Table 5:** The fitting results of species abundance distribution of chigger community.

Log Intervals Based on log_3_M	Individual Ranges of Chiggers in Each Log Interval	Midpoint Values of Each Individual Range	Actual No. of Chigger Species	Theoretical No. of Chigger Species
0	1	1	5	13.11
1	2–4	3	12	17.91
2	5–13	9	21	21.61
3	14–40	27	23	23.00
4	41–121	81	11	21.61
5	122–364	243	7	17.91
6	365–1093	729	12	13.11
7	1094–3280	2187	8	8.46
8	3281–9841	6561	13	4.82
9	9842–29,524	19,683	2	2.42
10	29,525–88,573	59,049	1	1.08

**Table 6:** Monthly variation in chigger infestation indexes on small mammal hosts.

Months	No. of Hosts		No. and *C_r_* of Chiggers		Infestation Indexes of Chiggers			Indexes of Chigger Community				
	Hosts Examined	Hosts Infested	No.	*C_r_*, %	*P_M_*	*MA*	*MI*	*S*	*R*	*H*	*E*	*D*
Jan.	110	78	11,801	5.42	70.91	107.28	151.29	50	5.23	2.13	0.55	0.18
Feb.	118	89	6416	2.95	75.42	54.37	72.09	32	3.54	2.24	0.65	0.13
Mar.	123	91	11,699	5.37	73.98	95.11	128.56	46	4.80	2.53	0.66	0.14
Apr.	104	51	2799	1.29	49.04	26.91	54.88	24	2.90	1.95	0.61	0.22
May	104	36	578	0.27	34.62	5.56	16.06	26	3.93	2.37	0.73	0.13
Jun.	108	40	2282	1.05	37.04	21.13	57.05	21	2.59	2.04	0.67	0.17
Jul.	109	63	4892	2.25	57.80	44.88	77.65	21	2.35	1.41	0.46	0.40
Aug.	114	93	45,750	21.02	81.58	401.32	491.94	21	1.86	0.93	0.31	0.65
Sep.	110	62	34,123	15.68	56.36	310.21	550.37	22	2.01	1.14	0.37	0.42
Oct.	102	99	39,568	18.18	97.06	387.92	399.68	24	2.17	1.19	0.37	0.46
Nov.	114	110	35,870	16.48	96.49	314.65	326.09	69	6.48	2.22	0.52	0.24
Dec.	113	110	21,893	10.06	97.35	193.74	199.03	52	5.10	2.52	0.64	0.11
Total	1329	922	217,671	100.00	69.38	163.79	236.09	115	9.28	2.55	0.54	0.18

Annotation: Species richness (*S*) and other community indices were calculated based on the identified chigger species, and 5454 unidentified chigger individuals were not included in the statistics.

## Data Availability

The original contributions presented in this study are included in the article. Further inquiries can be directed to the corresponding author.
